# Evidence-Based Management of Uterine Fibroids With Botanical Drugs-A Review

**DOI:** 10.3389/fphar.2022.878407

**Published:** 2022-06-22

**Authors:** Masita Arip, Vi Lien Yap, Mogana Rajagopal, Malarvili Selvaraja, K Dharmendra, Sasikala Chinnapan

**Affiliations:** ^1^ Allergy and Immunology Research Centre, Institute for Medical Research, Ministry of Health Malaysia, National Institute of Health Complex, Setia Alam, Malaysia; ^2^ Department of Pharmaceutical Biology, Faculty of Pharmaceutical Sciences, UCSI University, Cheras, Malaysia; ^3^ Narayan Institute of Pharmacy, Gopal Narayan Singh University, Jamuhar, India

**Keywords:** uterine fibroids, leiomyoma, botanical drug, polyherbal, mechanism, management

## Abstract

Uterine fibroids (UFs) are a common benign gynecological tumor that affect the majority of women over their lifetime. Several pharmacological agents are available to reduce the size of fibroids and ameliorate the symptoms of UF. However, these drugs are expensive and are usually associated with profound side effects. Thus, botanical drugs are gaining attention in this era due to their cost effectiveness with a comparable and more potent therapeutic efficacy while demonstrating lesser adverse effects. The objective of this review is to summarize the available information on the mechanism of various botanical drugs and polyherbal formulations with anti-uterine fibroid activity. A systematic search was performed on botanical drugs with anti-uterine fibroid activity using several search engines, which include PubMed, Google Scholar, and Science Direct. Based on the literatures identified, a total of five botanical drugs and three polyherbal formulations were included and discussed in this review, which yields useful information regarding the mechanism of different botanical drugs and polyherbal formulations in exerting anti-uterine fibroid activity for its potential use as an alternative treatment choice for uterine fibroids.

## Introduction

Uterine fibroids (UFs), also known as leiomyoma, are common benign gynecological tumors that proliferate from the myometrial smooth muscle cells into discrete masses (Ando et al., 2018). Generally, they affect 70–80% of women over their lifetime with the majority presenting without any symptoms (Baird et al., 2003). The clinical manifestations associated with UF include abnormal bleeding, pelvic pain, menorrhagia, infertility, recurrent miscarriages, and other obstetric-associated complications, which may lead to low quality of life. A cross-sectional study (Hervé et al., 2018) conducted among the French women reported that 64% of the participants had a moderate to significant decrease in their quality of life as a result of UF.

The incidence of UF was reported as 1.278% and 3.745% in Asia and African American women per year, respectively (Sheng et al., 2020). Moreover, studies also reported that black women have a higher lifetime prevalence of UF with more severe symptoms than white women (Stewart et al., 2013). US has reported that the cost involving uterine fibroids caused a profound impact on the nation and community, which requires a total of US$5.9 to $34.4 billion annually (Cardozo et al., 2012).

The etiology and/or the exact cause of UF are still unclear; however, genetics, cytokines, growth factors, hormones, such as estrogens and progesterone and/or their respective receptors, environmental and epigenetics, and the excessive synthesis of extracellular matrix (ECM), have been associated with the pathogenesis of UF (Flake et al., 2003; Ciavattini et al., 2013). In addition, epigenetics and microbiota were also associated with the development of UF (Li et al., 2003; Atkinson et al., 2006).

Although hysterectomy is the common and definitive treatment for UF, it is associated with several downsides, namely, the removal of the uterus and infertility. Generally, the treatment for UF depends on the size, location, symptoms, age, and fertility requirement of a patient. The available therapeutic treatment only gives temporary or partial relief from UF, whereas hormonal pills and NSAIDs produce serious side effects on the patients. Having these concerns, women are increasingly looking for an alternative option in treating fibroids.

In recent years, more research was conducted to identify natural extracts and botanical drugs for treating fibroids. With this in mind, we have conducted a detailed literature search to provide a general overview of the botanical drugs and polyherbal formulations available that may potentially treat and prevent uterine fibroids and their mechanism of action. Despite the fact that several reviews have been conducted on the use of botanical drugs on uterine fibroids, they mainly focused on the benefits and risks of using herbal or botanical drugs preparation (Liu et al., 2013; Fu et al., 2020) and on the effect of compounds on uterine fibroids (Li et al., 2020). This is the first review to our knowledge that discusses the detailed mechanisms of botanical drug extracts and polyherbal formulation in contributing to its anti-uterine fibroid activity.

## Methods

Well renowned and globally accepted scientific databases, namely, PubMed, Google Scholar, and Science Direct were searched systematically to obtain relevant references up to January 2022 using the term “herb,” “plant,” “medicinal plant,” and “polyherbal formulation” alone or paired with “leiomyoma,” ‘uterine fibroids,” and “hysteromyoma.” Only research articles written in English were included.

**FIGURE 1:**
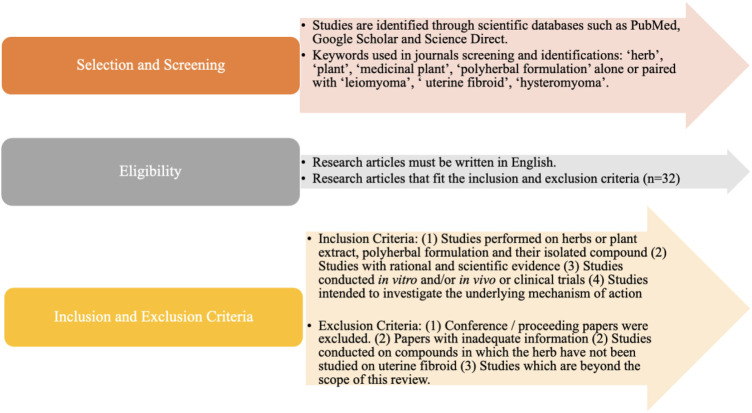
Schematic representation of a selection of articles.

The screening, eligibility, inclusion criteria, and exclusion criteria applied in the selection of the research articles were summarized in Figure 1. In this review, five botanical drugs and 3 polyherbal formulations have been identified based on the inclusion and exclusion criteria, with sufficient data available on their anti-uterine fibroid mechanisms.

A total of 32 articles were identified and deemed fit according to the inclusion and exclusion criteria. Among these articles, 4 studied *Curcuma longa* and/or its active constituents, 6 conducted on *Camellia sinensis* and/or its active constituents, 6 were on *Scutellaria barbata,* 2 were studied on *Euonymus alatus*, 1 study included both *Scutellaria barbata* and *Euonymus alatus*, 2 studies were conducted on the effect of *Fragaria x ananassa* on leiomyoma, 2 references were on Guizhi Fu Ling Wan (GZFLW), and 4 studies investigated on Lichong decoction. The rest of the studies were referenced in ‘5.3 Polyherbal *Sparganii rhizoma* (*Sparganium stoloniferum* (Buch.-Ham. Ex Graebn.) Buch.-Ham. Ex Juz [Typhaceae]) and *Curcumae rhizoma* (*Curcuma phaeocaulis* Valeton [Zingiberaceae] herb combination in Uterine fibroids.

## Drawbacks of the Current Treatments for Uterine Fibroids

Apart from surgeries such as hysterectomy and myomectomy, uterine artery embolization (UAE), and focused ultrasound surgery (FUS), both ultrasound- and magnetic resonance-guided focused ultrasound surgery (USgFUS and MRgFUS) are also the main treatment strategies for UF, with hysterectomy being the only definitive solution for UF. Hysterectomy is the surgical removal of the uterus and hence is not ideal for fertile women who intend to retain their uterus. In such a condition, myomectomy, UAE, or FUS will be an alternative option for women who wish to maintain their uterus intact. However, these surgical methods are limited to their complications and cost. With the knowledge that UF is hormone-dependent, pharmacological agents that target the hormonal pathways, such as gonadotropin-releasing hormone (GnRH) agonist, selective progesterone receptor modulator (SPRM), and aromatase inhibitor, have been developed as a treatment choice for UF.

GnRH agonist acts by causing an initial increase in the secretion of gonadotropin by binding to the GnRH receptor. Following that, GnRH agonist desensitizes the receptor, causing a decrease in gonadotropin release, and subsequently reduces the estrogen level secondary to the decrease in ovary stimulation by gonadotropin. In addition, GnRH agonist was also found to have a direct antiproliferative effect on the fibroid (Khan et al., 2010). Currently, GnRH agonists are approved by FDA to be used in the preoperative treatment of UF. However, its use is generally limited to 6 months due to the high incidence of side effects reported (Matsuo, 2004; DiVasta et al., 2007; Lethaby et al., 2017).

GnRH antagonist is the newer member in the class of GnRH analog following GnRH agonist. It acts by competitively occupying the GnRH receptors, thus reducing the level of estrogen and progesterone. Unlike GnRH agonist, it does not trigger the initial surge of follicle-stimulating hormone and luteinizing hormone. In 2020, FDA have granted approval for the use of elagolix, a GnRH antagonist in combination with estradiol and norethindrone acetate for the treatment of uterine fibroid (FDA, 2020). Relugolix, the latest GnRH antagonist is currently under clinical trial for the treatment of uterine fibroid and it offers the advantage of once-a-day dosing as compared to twice-daily dosing with elagolix (Al-Hendy et al., 2021). In 2019, relugolix has received approval in Japan for being marketed as a treatment for UF symptoms (Markham, 2019). However, similar to GnRH agonist, GnRH antagonist is associated with hypoestrogenic adverse effects, which require the addition of estradiol and norethindrone acetate (add-back therapy) to attenuate the hypoestrogenic effect (Schlaff et al., 2020). Due to the risk of bone loss and fractures, FDA does not recommend the use of elagolix for more than 2 years (FDA, 2020).

Selective progesterone receptor modulator (SPRM) is another class of compounds commonly used in UF, with a mixture of agonist and antagonist activity on the progesterone receptor. The two most commonly used SPRM are mifepristone (pure antagonist) and ulipristal acetate (UPA), which have been proven to be effective against UF. Initially, UPA have been used to reduce the fibroid volume for 3 months before surgery. However, recently, it is used in patients who refuse to remove their uterus (Pazzaglia et al., 2017; Rozenberg et al., 2017). Due to the progesterone antagonist activity of UPA on the endometrium, this could cause an unopposed estrogen activity, which may increase cell proliferation. Progesterone receptor modulator-associated endometrial changes (PAEC) have been found to occur in 0.4% of patients using UPA while 41%–78.8% of patients who developed PAEC appeared to be reversible upon discontinuing UPA (De Milliano et al., 2017). Recently, the European Medicines Agency (EMA) decided to suspend the use of UPA in treating UF due to the high incidence and severity of liver toxicity, with more than 900,000 women who were given with UPA for UF requiring liver transplant. Hence, to evaluate the overall safety with regards to the use of UPA, EMA has started a new review. No patient will be prescribed UPA for the first time during this period until the review has come to a conclusion (Ekanem and Talaulikar, 2021).

Aromatase inhibitor is used to block the action of aromatase, an enzyme that is responsible for the conversion of androstenedione to estrogen, resulting in increased cell proliferation and fibrosis. The two aromatase inhibitors that have been widely studied for UF are letrozole and anastrozole. In a randomized controlled trial comparing the effect of aromatase inhibitor and GnRH agonist on UF, it was found that both interventions are able to reduce the myoma volume and symptoms; however, aromatase inhibitors possess an advantage of rapid onset of action and the absence of the initial flare associated with GnRH agonist (Parsanezhad et al., 2010). The concern regarding aromatase inhibitor is the hypoestrogenic adverse effect, which includes hot flushes and bone loss. However, it has been reported that the use of aromatase inhibitors resulted in reduced hot flushes as compared to GnRH agonist, while the adverse effect of aromatase inhibitors are usually mild and occurs more frequently with prolonged use. Nevertheless, a Cochrane Systematic Review had concluded that the evidence to support the use of aromatase inhibitors in UF was insufficient (Song et al., 2013). A summary of the benefits and drawbacks of various surgical and pharmacological interventions for uterine fibroids is shown in Table 1.

**TABLE 1:** Possible benefits and drawbacks of surgical and pharmacological interventions.

Intervention	Benefits	Drawbacks
Gonadotropin-releasing hormone agonist (Matsuo, 2004; DiVasta et al., 2007; Yoo et al., 2007; Donnez et al., 2012; Han et al., 2013; Lee et al., 2017)	• Greater fibroid and uterine volume reduction than SPRM	
	• Increase preoperative hemoglobin	• Greater adverse effects, especially hot flushes and bone loss on prolonged use
	• Reduce blood loss during surgery	• Risk of disease recurrence
	• Fewer postoperative complications	
	• Uterus is preserved	
Gonadotropin-releasing hormone antagonist (Schlaff et al., 2020; Al-Hendy et al., 2021)	• Significant reduction in menstrual blood loss	• Risk of hot flushes, metrorrhagia and bone loss on prolonged use
	• Uterus is preserved	
	• Reduce uterine or fibroid volume	
	• Rapid symptoms improvement	
	• Do not trigger FSH and LH surge	
Selective Progesterone Receptor Modulator (SPRM) (Donnez et al., 2012; Kulshrestha et al., 2013; De Milliano et al., 2017; Lethaby et al., 2017)	• Less hot flushes than GnRH agonist	
	• Reduce uterine or fibroid volume	• Progesterone receptor modulator-associated endometrial changes
	• Increase preoperative hemoglobin	• Risk of disease recurrence
	• Reduce blood loss during surgery	
	• No hypoestrogenic side effects	
Aromatase Inhibitor (Parsanezhad et al., 2010; Brito et al., 2011; Song et al., 2013)	• Reduce fibroid size	• Bone loss on prolonged use
	• Uterus is preserved	
	• Fewer vasomotor adverse effect than GnRH agonist	
	• Rapid onset of action than GnRH agonist	
Levonorgestrel-intrauterine device (Grigorieva et al., 2003; Sayed et al., 2011; Socolov et al., 2011; Jiang et al., 2014; Dhamangaonkar et al., 2015)	• Most effective for reducing blood loss	• Irregular bleeding
	• Reduce fibroid and/or uterine volume	• Increase risk of device expulsion
Hysterectomy (Pitter et al., 2014; Aarts et al., 2015)	• Definitive treatment	• Increase risk of blood loss, postoperative fever, and surgical site infection
	• Improved quality of life and satisfaction	• Uterus is removed
Myomectomy (Yoo et al., 2007; Shiota et al., 2012; Kongnyuy and Wiysonge, 2014)	• Able to preserve fertility	• Risk of myoma recurrence
	• Lower rate of complication	• Risk of bleeding
Uterine artery embolization (Moss et al., 2011; Gupta et al., 2012; Jun et al., 2012; Lethaby and Vollenhoven, 2015; Sandberg et al., 2018)	• Minimally invasive	
	• Shorter duration of hospitalization	• Higher risk of minor complication
	• Shorter recovery time	• Risk of myoma recurrence and reintervention
	• Less major complications	
Focused ultrasound surgery (USgFUS and MRgFUS) (Stewart et al., 2007; Gorny et al., 2011; Chen et al., 2018; Wong, 2020)	• Noninvasive	• Risk of skin burn, weakness, or numbness of the lower limbs
	• Shorter duration of hospitalization	• Risk of myoma recurrence and reintervention
	• Less morbidity	

The table clearly summarizes that the availability of cheaper, safer, and more effective alternative treatments for uterine fibroids could greatly benefit the society and enhances their quality of life. There has been an improvement and escalated research growth that attempts to discover natural alternatives that could be used as an anti-UF agent. Botanical drug products have an increased demand in this era due to their lower cost with a comparable or more potent therapeutic efficacy while being associated with lesser adverse effects. This review will, therefore, discuss the mechanisms of action of various natural botanical drugs and herbal formulations that have been studied against UF *in vitro*, *in vivo,* and in clinical trials. A total of five botanical drugs and 3 polyherbal formulations have been included and summarized.

**FIGURE 2:**
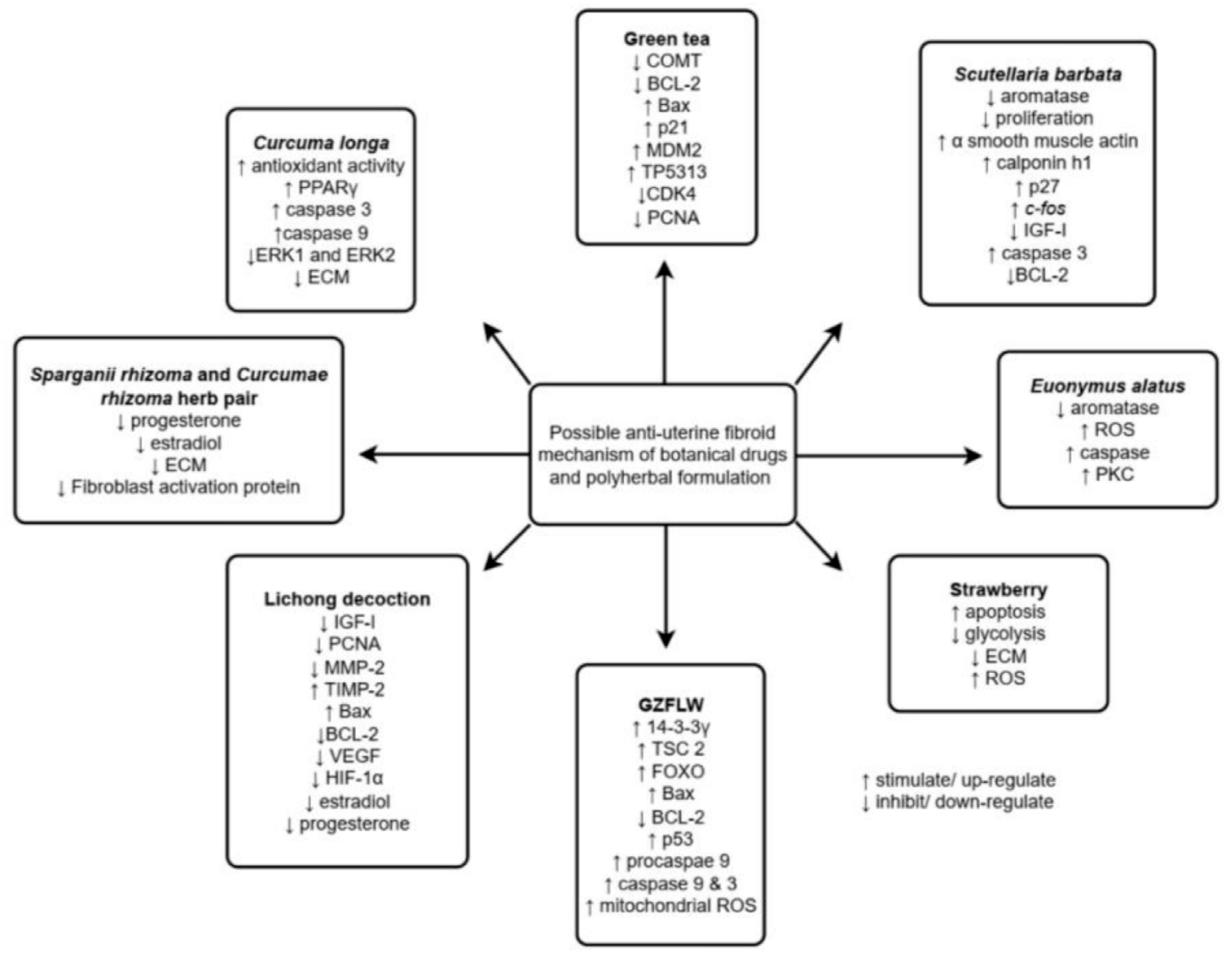
Summarized anti-uterine fibroid mechanisms of various botanical drugs and polyherbal formulations. Adapted from (Islam et al., 2013).

Figure 2 shows the summarized information regarding the mechanism of various botanical drugs and polyherbal formulations in exerting anti-uterine fibroid activity.

## Botanical Drugs With Anti-Uterine Fibroid Activity

### *Curcuma longa* L [Zingiberaceae] as Alternative Treatment for Uterine Fibroids

*Curcuma longa*, commonly known as turmeric is a rhizomatous herbaceous perennial herb that belongs to the ginger family and is planted extensively throughout Asia, India, and other tropical countries. It has been used since ancient times for flavoring and coloring purposes due to its yellowish nature. In the context of Traditional Chinese Medicine (TCM), *Curcuma longa* was included as one of the herbs in the tumor-shrinking decoction, which is currently under phase-III clinical trials (ClinicalTrials.gov, 2014; Cheng et al., 2019). *Curcuma longa* was also added to tonify qi as UF is believed to be a product of vital energy deficiency and stasis of blood according to TCM theories (ClinicalTrials.gov, 2014). Likewise, *Curcuma longa* is also used in Ayurveda as the main ingredient for the formulation *Haridra Khanda*, which appeared to be effective in relieving the symptoms and size of UF in a case series (Dhiman, 2014).

The ability of turmeric extract in protecting the uterine myometrium from oxidative damage-induced UF has been reported (Eze-Steven, 2019). The reactive oxygen species (ROS) is able to induce the proliferation of leiomyoma smooth muscle cells and it is also necessary for triggering the MAPK1/MAPK3 signaling pathway, which contributes to leiomyoma smooth muscle cell proliferation (Mesquita et al., 2010). Thus, the antioxidant potential of turmeric and its protective effect on uterine myometrium shows potential to be used as a treatment or prevention for UF.

The antioxidant activity may be attributed to curcumin, the main polyphenol and active compound present in *Curcuma longa* which has the ability to increase the level of antioxidant enzymes, which include catalase, superoxide dismutase, and glutathione peroxidase (Panahi et al., 2012; Lin et al., 2019). Up to date, the anti-uterine fibroid activity of curcumin has been evident in many literatures. The size, volume, and proliferative activity of UF were significantly reduced when patients took 1.2 g of curcumin orally every day for 6 months, with no adverse effects observed (Sukonthanonta, 2015). Ali and Laila (Ali and Ali, 2013) reported a decrease in uterine and myoma volume followed by the significant increase in hemoglobin percentage in patients taking 1.35 g of curcumin daily for 3 months was consistent with the previous study with no side effects being reported. However, the interpretation of results from both of the studies was restricted due to a lack of information on control groups.

*In vitro* study with curcumin on Eker rat-derived uterine leiomyoma cell lines (ELT-3) showed the ability of curcumin to inhibit leiomyoma cell proliferation while increasing apoptosis by acting as a PPARγ ligand (Tsuiji et al., 2011). The exact signaling pathway of PPARγ in UF has not been clearly characterized despite the fact that PPARγ protein isoforms are expressed at higher levels in UF (Tsibris et al., 1999) or a relatively equal level as compared to the normal myometrium (Houston et al., 2003). Among the several mechanisms proposed in attributing the ability of PPARγ ligand in inhibiting leiomyoma cell growth, Houston suggested that the inhibition was mediated by negative cross-talk between ER and PPAR signaling pathway where the activation of PPARγ inhibits ER-mediated gene expression (Houston et al., 2003). Other possible mechanisms include the accumulation of Fas(Nam et al., 2007), downregulation of cyclooxygenase-2 (COX-2), and caspase-3 activation (Liu et al., 2007).

Apoptosis of uterine leiomyoma cells was also stimulated by the expression of caspase-3, an important mediator for apoptosis, and by caspase-9, an initiator of apoptosis (Malik et al., 2009). On top of that, ERK1 and ERK2 proteins were also decreased in a concentration-dependent manner with curcumin (Malik et al., 2009). These proteins are involved in the Ras/Raf/MEK/ERK signaling pathway which plays a role in cellular proliferation and survival and is involved in the pathophysiology of UF. Supporting this, ERK1 and ERK2 are overexpressed in leiomyoma as compared to the normal myometrium (Yu et al., 2008). Knowing the fact that UF is hormone-dependent, which is mainly associated with the estrogen hormone (Marshall et al., 1998), estradiol has been found to contribute to the growth of leiomyoma via the activation of ERK (Nierth-Simpson et al., 2009). Hence, a reduction of this protein can largely contribute to the anti-uterine fibroid activity.

Leiomyoma is a benign tumor with excessive ECM production. The ability of curcumin to down-regulate the expression of fibronectin, one of the ECM proteins, which is overexpressed in leiomyoma as compared to normal myometrium, has also been documented (Malik et al., 2009). The significant inhibitory effect of curcumin on the expression of ECM receptors in non-small-cell lung cancer (Li et al., 2017) and reducing the activity of MMP-2 and up-regulating TIMP activity in various cancer cells (Mitra et al., 2006; Hassan and Daghestani, 2012; Supriono et al., 2018) suggest the possible mechanism of curcumin in producing anti-uterine fibroid activity via reducing the ECM production, thus further study investigating the effects of curcumin on various ECM-related receptors and proteins in leiomyoma cells may be conducted.

### *Camellia sinensis* (L.) Kuntze [Theaceae] (Green Tea) in Uterine Fibroids

A randomized controlled trial reported on the effect of green tea extract [45% epigallocatechin gallate (EGCG)] in women with symptomatic UF showed a significant reduction in the fibroid volume as compared to the placebo group after taking 800 mg of green tea extract for the duration of 4 months. The severity of symptoms associated with fibroid, average blood loss and HRQL were also significantly improved with the use of green tea extract as compared to placebo, with no adverse effects identified (Roshdy et al., 2013). The ability of green tea to exert anti-UF activity is mainly attributed by epigallocatechin gallate (EGCG), one of the main active constituents in green tea, which constitutes more than 40% of the total polyphenol of green tea catechin and is present at about 142 mg in a 200 ml of green tea (Yang and Wang, 1993). Numerous studies have identified the antiproliferative and apoptosis induction effect of EGCG on uterine fibroid cells. Although the exact mechanisms and pathways involved are unclear, a combination of mechanisms has been proposed in respective to this.

Catechol-O-methyl transferase (COMT) is an enzyme expressed at a higher level in leiomyoma as compared to the normal myometrium (Salama et al., 2006). COMT is involved in converting 2-hydroxyestradiol (an antiestrogen) to 2-methoxyestradiol (proestrogen), creating a hyper-estrogenic milieu due to the decrease in antiestrogen and an increase in proestrogen. Knowing UF is largely estrogen-dependent, the increased estrogenic environment has a great potential to increase UF proliferation and growth. In addition, those who have COMT Val/Val genotype (genotype with greater enzyme expression) were associated with a significantly increased risk of UF as compared to those with Val/Met genotype (intermediate expression) and Met/Met genotype (low expression), which could have explained the higher risk of UF in African American women (47% having Val/Val genotype) (Al-Hendy and Salama, 2006). *In vitro* study showed that the ability of green tea extract in inhibiting the proliferation of leiomyoma cells is in part due to the potential of EGCG in reducing the expression of the COMT enzyme (Zhang et al., 2014). Be that as it may, *in vivo* study showed no impairment of EGCG on COMT activity and even increase by 24% after consumption of EGCG (Lorenz et al., 2014). Hence, further study is warranted to confirm the antiproliferative effect of EGCG on leiomyoma via the COMT pathway.

Decreased BCL-2 protein and elevated Bax protein expression are other mechanisms leading to apoptosis by EGCG (Zhang et al., 2010a). BCL-2, the proto-oncogene, has the ability to block apoptosis, leading to reduced cell death and an enhanced proliferation (Omar et al., 2019). Bax protein is a proapoptotic member that acts by forming pores on the mitochondrial outer membrane, allowing cytochrome c to translocate into the cytoplasm, resulting in the loss of energy production and the activation of proteolytic cascade leading to apoptosis (Westphal et al., 2014). It was reported that EGCG significantly up-regulated BAX protein and down-regulated BCL-2 protein in a dose-dependent manner when EGCG was tested in HuLM cells. The p53 pathway genes, namely, BAX, p21, MDM2, and TP5313, which are involved in DNA repair, apoptosis, and cell cycles were also up-regulated with EGCG treatment (Zhang et al., 2010a).

The EGCG could have also exerted cell cycle arrest by causing a significant decrease in the expression of CDK4 and PCNA (Zhang et al., 2010a; 2010b). Complex formation between cyclin and CDK4 is an important driver in the cell cycle through the G1 phase to S phase transition. The reduced expression of CDK4 by EGCG, blocks the transition from G1 phase to S phase, which could cause a G0/G1 phase arrest of the cell cycle. On the other hand, PCNA is essential for cell proliferation, and it interacts with various proteins in regulating the cell cycle, DNA replication, and DNA repair process (Mansilla et al., 2020).

BMP2 gene appeared to have a 14-fold greater expression upon EGCG treatment on HuLM cells (Zhang et al., 2010a). BMP2 is a member of the TGF-β superfamily, which plays a vital role in the regulation of cell proliferation, differentiation, and apoptosis. A recent study on colorectal cancer demonstrated the role of BMP2 in reducing growth, enhancing apoptosis, and decreasing the development of tumors *in vivo* (Vishnubalaji et al., 2016), supporting that the decreased expression of BMP2 also leads to the progression of prostate cancer (Horvath et al., 2004). These data, therefore, propose the possibility of BMP2 as a potential target choice for UF treatments.

EGCG was also identified to differentially inhibit TNF-α and LPS-mediated activation of NF-κB (Ahmad et al., 2000), and also reduce the expression of bcl2A1 (Zhang et al., 2010a), the key factor of NF-κB pathway. NF-κB is a transcription factor involved in promoting angiogenesis, cell proliferation, and inhibiting apoptosis and is often activated in cancer cells (Taniguchi and Karin, 2018). Noteworthy, EGCG only reduces cell proliferation and induces apoptosis in cancer cells but not in normal cells, demonstrating its invulnerability/safeness to be used as a potential alternative treatment (Ahmad et al., 2000).

Apart from producing promising effects, EGCG has several drawbacks, such as low stability, poor bioavailability, and highly metabolized under physiological conditions. Taking these drawbacks into consideration, the prodrugs of EGCG analogs were synthesized with the compounds identified as 2a and 4a having lower susceptibility to be metabolized or inhibited by COMT and having an amplified antiproliferative, antiangiogenic, and antifibrotic effect in HuLM (Ahmed et al., 2016).

### *Scutellaria barbata* D. Don [Lamiaceae] for Uterine Fibroids

*Scutellaria barbata* (SB) is a perennial herb, known in TCM, and traditional Korean Medicine as “Ban-Zhi-Lian” and “Ban-Ji-Ryun,” respectively. This botanical drug exhibits anti-inflammatory, antitumor activity, and antimutagenic effects. Its effects on UF were established by several studies with multiple mechanisms of action being reported.

In 2004, aromatase inhibitory activity of SB in leiomyoma cells was reported and the enzyme was inhibited in a time- and dose-dependent manner (Lee et al., 2004b). Aromatase is an enzyme responsible for converting androstenedione to estrogen. With the ability of SB to inhibit intracellular aromatase activity in leiomyoma cells, it potentially reduces the production of estrogen in the fibroids and thus reduces the stimulation of estrogen in causing cell proliferation and subsequently fibrosis or increased size of the fibroid.

SB also exhibited a dose-dependent inhibition on the proliferation of leiomyoma and normal myometrial cells. SB increases the fraction of cells in the G1 phase of the cell cycle and was suggested to exhibit its antiproliferative action via blocking the transition of the cell cycle from the G1 phase to the S phase or by arresting the cell cycle at the G1 phase (Lee et al., 2004d). In addition, the expression of smooth muscle cell differentiation markers, which include α-smooth muscle actin, calponin h1, and cell cycle inhibitor p27, in both leiomyoma and normal myometrial cells was increased after treatment with SB. On the contrary, SB has no effect on cyclin E and cdk2, which are gene products associated with G1 phase of the cell cycle (Lee et al., 2004d). P27 protein is considered a potent tumor suppressor, which is able to induce apoptosis and reduce the viability and proliferation of UF cells (Ramachandran et al., 2008). With this, it is possible that SB induces its antiproliferative effect via an induced differentiation of smooth muscle cells and an up-regulation of p27.

When human uterine leiomyoma cells were being treated with SB, it was observed that *c-fos* mRNA expression was being induced by SB via the cAMP/PKA pathway which was activated by the increased in cAMP level (Lee et al., 2004a). This was evident by the fact that the PKA inhibitor inhibited the SB-induced *c-fos* gene expression (Lee et al., 2004a). *c-fos* is a proto-oncogene that is closely involved in cell growth and differentiation and was also suggested to be involved in smooth muscle cell differentiation (Raimundo et al., 2009). *c-fos* gene expression has been identified to be significantly lower in leiomyoma cells as compared to normal myometrium which is consistent with the previous study that has suggested that a decrease in the smooth muscle cells progenitors could have contributed to the formation of UF (Arslan et al., 2005). Hence, further studies are needed to determine whether the increased *c-fos* expression in leiomyoma cells induced by SB is associated with its antiproliferative actions (Lee et al., 2004a).

The higher incidence of UF and the greater fibroid size during the first trimester of pregnancy have suggested the role of hCG in the pathogenesis of UF (Benaglia et al., 2014; Ciavattini et al., 2016). Supporting this, hCG significantly increases the proliferation in both leiomyoma and normal myometrial cells. In this context, SB demonstrated an ability to reduce the proliferating effect of hCG in leiomyoma and myometrial cells. On top of that, treatment with SB also reduces the expression of PCNA, cyclin E, and cdc2 in hCG-treated leiomyoma cells (Lee et al., 2004c).

SB has also been observed to down-regulate the expression of IGF-I in leiomyoma cells which were overexpressed in UF as compared to normal myometrium (Párrizas et al., 1997). IGF-I functions as a survival factor that is able to block apoptosis in a number of cell types; therefore, the over-expression of IGF-I has been associated with its tumorigenic activity, protecting the cancer cells from apoptosis (Párrizas et al., 1997). Studies have also demonstrated the role of IGF-I in promoting mitosis of cells, and its ability to particularly promote the uterine smooth muscle cells proliferation, therefore making it a potential contributor to the formation of UF (Maruo et al., 2007; Baird et al., 2009). Besides the aforementioned mechanisms, SB was also reported to exhibit its antiproliferative effect and apoptosis inductive effect by causing the release of cytochrome c from mitochondrial into the cytosol, with a subsequent increase in caspase-3-like activity and causing a decrease in BCL-2 protein in leiomyoma cells, all of this contributing to its anti-uterine fibroid activity (Lee et al., 2006; Kim et al., 2008).

### *Euonymus alatus* (Thunb.) Siebold [Celastraceae] for Uterine Fibroids

*Euonymus alatus* (EA) is a medicinal plant used for the treatment of tumors in TCM and traditional Korean medicine. Similar to *Scutellaria barbata*, *Euonymus alatus* is also able to inhibit the aromatase enzyme activity and was found to be 10 to 30 times more potent as compared to SB. EA exhibited a time- and dose-dependent inhibition of intracellular aromatase activity in leiomyoma cells, which potently inhibit the capability of the tumor cell in self-supplying estrogen, the hormone that results in the proliferation of leiomyoma cells (Lee et al., 2004b). EA was observed to induce apoptosis by acting as a prooxidant and induced the release of cytochrome c from mitochondria, followed by the activation of caspase in leiomyoma cells. Interestingly, the apoptotic effect of EA was observed only in leiomyoma cells without any effect on the peripheral blood mononuclear cells, which greatly reduces the possibility of causing an adverse effect (Kim et al., 2006). EA also demonstrated the ability to increase PKC activity in uterine leiomyoma cells and a slight increase in the normal myometrial cell. The activity of PKC is lesser in leiomyoma cell as compared to normal myometrium; however, the exact role of PKC in the pathogenesis of UF is not well established as of now (Lee et al., 2005). Even so, it was proposed that PKC could be involved in the downstream pathway of TGF-beta2, which is known to contribute to fibroid development (Laping et al., 2007).

### *Fragaria x Ananassa* (Duchesne Ex Weston) Duchesne Ex Rozier [Rosaceae] (Strawberry) as Alternative Treatment for Uterine Fibroids

In recent years, strawberry extract has also exhibited potential as a therapeutic and/or preventive agent against UF. Islam *et al.*(Islam et al., 2017) discovered that anthocyanin-rich strawberry extract is able to induce apoptosis, inhibit glycolysis, and significantly reduce ECM components, namely, collagen 1A1, fibronectin, and versican in leiomyoma cells. The author has suggested that the possible mechanism at which strawberry extract induces apoptosis is due to the increased ROS production. It is worth noting that the ROS production and the percentage of apoptotic and dead cells are significantly higher in leiomyoma cells; however, the ROS production was decreased instead while there was no significant difference in the percentage of apoptotic and dead cells in the normal myometrial cell, which could have indicated the possibility of strawberry extract to only target the UF cells while maintaining a homeostatic condition in the normal myometrial cells, hence lesser side effect could be achieved with the use of the strawberry extract.

Activin A, a member of the TGF- β superfamily was thought to be involved in the pathogenesis of UF by increasing the ECM components, such as versican, fibronectin, and collagen 1A1, at least in part through activating the Smad-2/3 and p38-MAPK signaling pathway (Islam et al., 2014; Bao et al., 2018). The treatment of the *Alba* cultivar of the strawberry extract was found to inhibit the induction of collagen 1A1, fibronectin, and versican mRNA expression by activin A in leiomyoma cells (Islam et al., 2017)*.*

Following that, the same group of researchers investigated the anti-UF activity of five different cultivars of strawberry. It was identified that Romina followed by the Alba cultivar produced the most promising anti-UF activity, which suggests its potential in using strawberry extract as an alternative treatment for UF (Giampieri et al., 2019).

## Polyherbal Formulation for Uterine Fibroids

It has been observed that the etiology and pathophysiology of uterine fibroid are multifactorial and the apoptosis and reduction in fibroid volume and size can be achieved via several pathways. Hence, to obtain a multimodal activity in the management of uterine fibroid, certain purposeful mixtures of herbs have been evaluated on their effect on the uterine fibroid. Such a polyherbal is able to target different pathways and pathological events from several approaches and potentially provide a more effective anti-uterine fibroid activity while improving the quality of life of the patients. In this context, we have reviewed several polyherbal formulations that have been studied on leiomyoma cells, with their anti-uterine fibroid mechanism being described.

### Polyherbal GZFLW in Uterine Fibroids

Guizhi Fu Ling Wan (GZFLW) is a famous traditional Chinese herbal formula and is the most frequently prescribed traditional Chinese formula in Taiwan (Yen et al., 2015). GZLFW is a formula consisting of five herbs in the ratio of 1:1:1:1:1 (g/g), namely Cinnamomi Ramulus (the dried twig of *Cinnamomum cassia* (L.) J. Presl), Poria (the dried sclerotia of *Poria cocos* (Schw.) Wolf), Moutan Cortex (the dried root bark of *Paeonia x suffruticosa* Andrews [Paeoniaceae]), Persicae Semen (the dried mature seed of *Prunus persica* (L.) Batsch [Rosaceae]) and Paeoniae Radix Alba (the dried root of *Paeonia lactiflora* Pall [Paeoniaceae]) (Xiao et al., 2012). Several studies that have investigated the effect of GZLFW on leiomyoma cells found that GZLFW is able to reduce cell proliferation and viability in a dose-dependent manner (Shen et al., 2016; Lee et al., 2019). In addition, a meta-analysis revealed that the combination of GZLFW with mifepristone was more effective in reducing the fibroid volume as compared to mifepristone alone. GZFLW is also able to improve the symptoms of dysmenorrhea in UF patients with no serious adverse effects being found (Chen et al., 2014).

The mechanism of GZFLW in inhibiting the proliferation and the induction of apoptosis has not been clearly defined; however, several possible mechanisms have been suggested. 14-3-3γ protein is a phosphoserine or phosphothreonine binding protein that is involved in various cellular processes, such as cell proliferation, apoptosis, and cell cycle (Morrison, 2009). 14-3-3γ is able to associate with FOXO and TSC2, preventing them from being dephosphorylated which controls the transcription of cytoplasmic and nuclear proteins subsequently affecting cell proliferation and apoptosis (Morrison, 2009; Khorrami et al., 2017). GZFLW is able to significantly increase the expression of 14-3-3γ, TSC2, and FOXO in human primary uterine leiomyoma cells as compared to the nontreatment group, which suggests the possible pathway of GZFLW in inhibiting proliferation and inducing apoptosis (Shen et al., 2016). Although 14-3-3γ signal transduction pathway has been associated with the formation of several cancer, its role in uterine fibroid formation is not well understood. However, the significant downregulation of 14-3-3γ identified in UF as compared to the normal myometrium suggests its role in the origin or growth of UF (Lv et al., 2008; Wang et al., 2012).

As mentioned earlier, Bax and BCL-2 play an opposite role in the regulation of apoptosis and thus the ratio of Bax to BCL-2 is often used as an indicator for apoptosis. GZFLW was observed to increase the ratio of Bax to BCL-2 in a dose-dependent manner and at the same time up-regulating the tumor suppressor gene p53 (Lee et al., 2019). The p53 mediates apoptosis by inducing the release of mitochondria cytochrome c with subsequent caspase activation along with the cleavage of caspase substrates. Following the release of cytochrome c into the cytosol, with the presence of ATP, it facilitates the activation of caspase-9 which then activates caspase-3 and the other effector caspases (Schuler et al., 2000). Besides affecting Bax, BCL-2, and p53, GZFLW exhibits a dose-dependent increase in the expression of procaspase-9, cleaved-caspase-9, and cleaved-caspase-3 (Lee et al., 2019).

GZFLW is also able to induce mitochondrial ROS production in human uterine leiomyoma cells (Lee et al., 2019). Mitochondrial ROS plays a critical role in apoptosis. A high level of mitochondrial ROS can lead to the release of mitochondrial apoptogenic factors, such as cytochrome c, which initiates intrinsic apoptosis (El-Osta and Circu, 2016). All these mechanisms, therefore, show that GZFLW induces apoptosis via the mitochondria-intrinsic mechanism.

### Polyherbal Lichong Decoction in Uterine Fibroids

Lichong decoction (LD) is another Chinese herbal formulation that has been studied for its effect on UF. The herbs included in this decoction includes 9 g of the root of *Astragalus mongholicus* Bunge [Fabaceae]*,* 6 g of root of *Codonopsis pilosula* (Franch.) Nannf [Campanulaceae]*,* 6 g of rhizome of *Atractylodes macrocephala* Koidz [Asteraceae], 15 g of rhizome of *Dioscorea opposita* Thunb [Dioscoreaceae]*,* 12 g of root of *Trichosanthes kirilowii* Maxim [Cucurbitaceae]*,* 12 g of rhizome of *Anemarrhena asphodeloides* Bunge [Asparagaceae], 9 g of rhizome of *Sparganium stoloniferum* (Buch.-Ham. Ex Graebn.) Buch.-Ham. Ex Juz [Typhaceae**]** *,* 9 g of rhizome of *Curcuma phaeocaulis* Valeton [Zingiberaceae] and 9 g of *Endothelium Coreneum Gigeriae* (chicken gizzard)*.* This decoction was traditionally formulated for the treatment of UF by strengthening the healthy *Qi,* enhancing blood flow, and eliminating disease-causing pathogens (Wang et al., 2016).

In recent studies, several mechanisms have been proposed for the ability of LD to be used as a treatment of UF. According to Li *et al.*, it was found that when LD is used on UF-induced rats, the expression of IGF-I and PCNA mRNA were significantly reduced as compared to the model group, suggesting its ability to reduce leiomyoma cell proliferation via this mechanism. The cells appeared ordered with the organelles appearing almost normal under a transmission electron microscope with the collagen fibers arranged relatively regularly as compared to the model control group where the collagen fibers were irregular and disordered with the organelles being malformed (Li et al., 2012). In line with the effect of LD on the collagen fibers, this group, later on, discovered that the MMP-2 protein expression was significantly reduced in rats treated with LD with an increased tissue inhibitor of metalloproteinase-2 (TIMP-2) expression, suggesting the influence of LD on the ECM (Wang et al., 2016). When there is a change in the ECM component, it will affect the stiffness and thus affect the development of fibroid. In the normal myometrium, homeostasis is achieved with the assistance of matrix metalloproteinases (MMP), which are involved in remodeling and degrading certain constituents of the extracellular matrices, such as collagen, which contributes to the stiffness of ECM (Curry and Osteen, 2003). Studies throughout the years have proved that the activity of MMP-2 is significantly higher in UF as compared to control (Bodner-Adler et al., 2004; Bogusiewicz et al., 2007a; Korompelis et al., 2015). MMP-2 mediates the degradation of collagen type IV and other components of ECM consequently, interfering with differentiation and proliferation (Moiseeva, 2001; Bogusiewicz et al., 2007b). On the other hand, TIMP-2 plays an opposite role to MMP-2 by inhibiting the protease activity in tissues undergoing remodeling of the ECM (Edelstein, 2017), thus a balance between MMP-2 and TIMP-2 plays a key role in maintaining the proper development and metabolism of the ECM. The ability of LD in reducing MMP-2 and increasing TIMP-2 expression, therefore, suggests that it may play a role in the protection of the uterus, and may also play a preventive and treatment role in UF.

In addition, when the UF animal model was being treated with LD the BCL-2 expression in the uterine tissue was significantly decreased with an increase in Bax mRNA expression, thus showing the apoptotic potential of LD in the treatment of UF (Li et al., 2013). LD was also proposed to reduce the size of the uterine in UF by suppressing angiogenesis via the reduction of VEGF expression and the downregulation of HIF-1α. The level of estradiol and progesterone, which are associated with the pathogenesis of UF, was also lowered with high-dose LD (Wang et al., 2020). Regardless of the promising effect of Lizhong decoction as proposed by the literatures, it is worth noting that the literatures do not provide the full botanical taxonomic names for the individual herb present in the formulation, while the common names given can be misidentified with the presence of various species and family available for the same herb as with the name provided.

### Polyherbal *Sparganii rhizoma* (*Sparganium stoloniferum* (Buch.-Ham. Ex Graebn.) Buch.-Ham. Ex Juz [Typhaceae]) and *Curcumae rhizoma* (*Curcuma phaeocaulis* Valeton [Zingiberaceae] Herb Combination in Uterine Fibroids

Another well-studied traditional Chinese herb is *Sparganii rhizoma*, which is one of the most frequently prescribed herbs and *Curcumae rhizoma.* There were studies that have been conducted on the effect of this herb pair on UF rats. When *Sparganii rhizoma* was studied in mice, it was observed that there was a significant decrease in the fibroblast growth factor-1 (FGF-1) and VEGF level, suggesting that *Sparganii rhizoma* may have an effect on angiogenesis (Sun et al., 2011). On the other hand, studies have shown and proven the antiangiogenic activity of *Curcumae rhizoma* essential oil and also its ability in inhibiting cell proliferation. In addition, as mentioned previously that MMP-2 was significantly increased in leiomyoma cells, *Curcumae rhizoma* was also reported to inhibit the expression of MMP-2 which could also contribute to its use in the treatment of UF (Chen et al., 2011). The essential oil from *Curcumae rhizoma* was also able to induce apoptosis by decreasing the level of BCL-2 and at the same time inhibit the phosphorylation of the AKT/NF-κB pathway (Chen et al., 2013).

When both of these herbs are used together (known as CRSR, CR:SR = 1:1) in the treatment of UF, the uterine mass was found to be decreased significantly with a significantly lowered progesterone and estradiol level. Moreover, CRSR was found to affect various ECM-associated genes, which may be responsible for its ability to decrease uterine mass (Yu et al., 2019). In line with the hypothesis that CRSR produces an effect on the ECM, another independent study reported a reduced expression of fibroblast activation protein, which is a collagen component of the ECM and TGF-b (Feng et al., 2021).

## Future Prospect

At present, although conventional pharmacological agents used for uterine fibroid continue to exhibit numerous drawbacks, it is still regarded as the ultimate choice of treatment for patients who prefer a noninvasive approach. Natural herbs and botanical drug products which have been widely reported *in vitro* showed favorable outcomes in the treatment of uterine fibroid. However, there are certain limitations that hindered its application in clinical practice. It is important to take into consideration that many phytoconstituents have a low bioavailability when they are consumed orally as the body considered them xenobiotics (Karaś et al., 2017) and, thus, may produce a significantly different result as that demonstrated from *in vitro* studies. In addressing the bioavailability issue, several novel delivery systems incorporating natural herbs or compounds have been developed (Zheng et al., 2019; Wang et al., 2021). Accordingly, more clinical studies are needed to determine their safety and efficacy in a well-designed clinical trial before they can be introduced and incorporated into clinical guidelines for the treatment or prevention of uterine fibroids.

**FIGURE 3:**
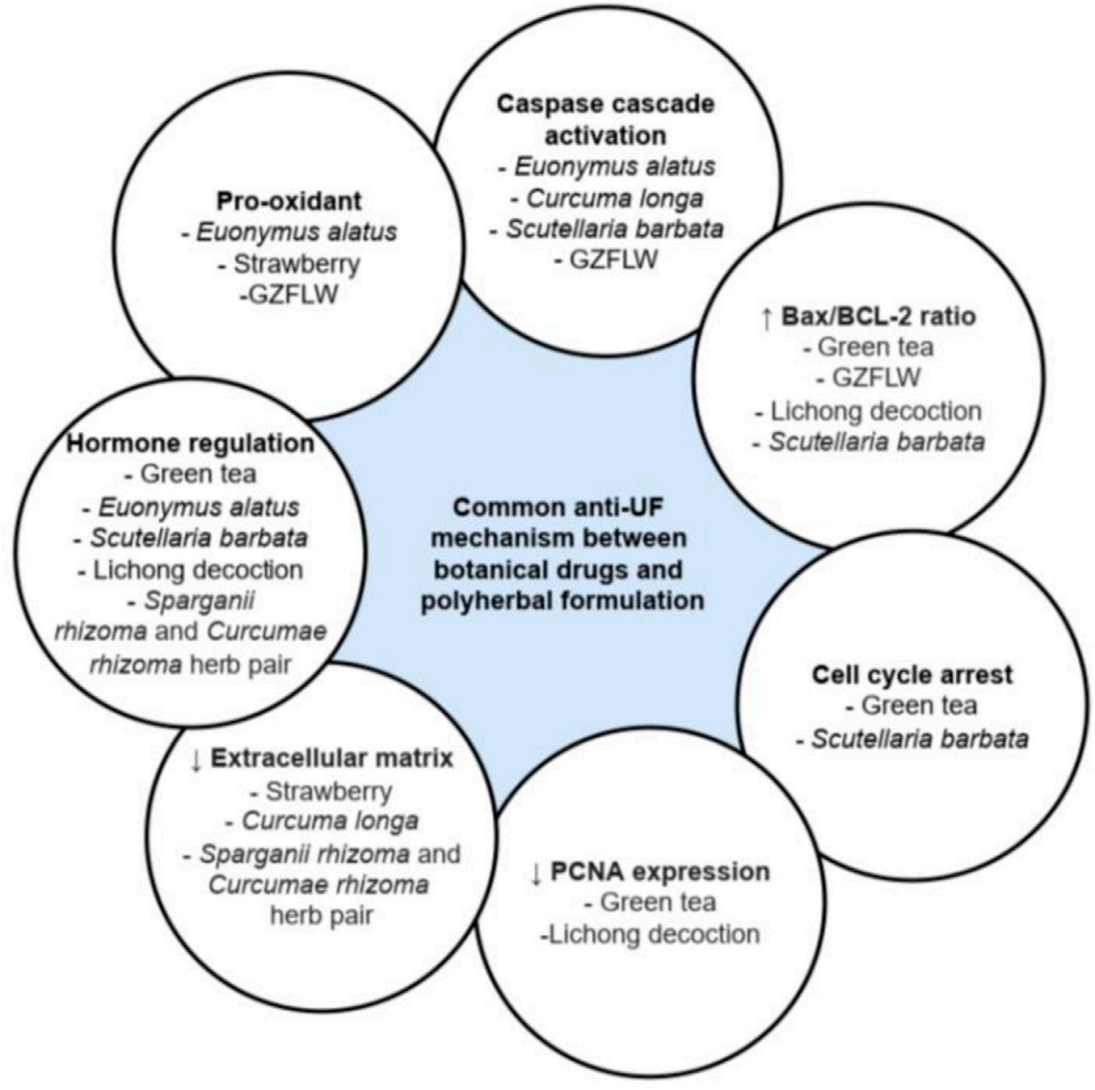
Common anti-uterine fibroid mechanism between botanical drugs and polyherbal formulation.

Among the botanical drugs and polyherbal formulations reviewed, only *Curcuma longa* (Ali and Ali, 2013; Sukonthanonta, 2015)*, Camellia sinensis* (Roshdy et al., 2013), and GZFLW (Chen et al., 2014) have been investigated in clinical trials on their effect against UF. Among these, the clinical studies against *Curcuma longa* were limited without the presence of a negative control group, while a systematic review of randomized clinical trials conducted on GZFLW revealed that the trials have a high risk of bias, with no description of the allocation concealment of the participants. Hence, a well-designed clinical trial should be conducted in the future before these botanical drugs can be recommended for the treatment of UF. In addition, different botanical drugs may exert anti-UF activity via the same mechanism, as shown in Figure 3. Thus, combining the different botanical drugs to promote synergistic effect via the same or different pathway can be considered and investigated. It is also possible for future studies to investigate the efficacy and safety of using botanical drugs and conventional pharmacological agents in combination, which may allow the dose of the pharmacological agents to be reduced, subsequently reducing the adverse effects experienced by the patients. The inclusion of only articles acquired from online platform and in English only presents limitation in this review. However, most of the recently published articles that fit the inclusion and exclusion criteria were all reviewed comprehensively and included in the present review.

## Conclusion

The use of botanical drugs as a part of treatment has been a practice since ancient times, and it is being imposed in this era with the urge of evaluating their mechanism of action and their associated safety profile. Many drugs available in the market are derived from plants and play an important role in today’s modern medicine. The ideal pharmacological agent for UF should be effective and affordable, with a short treatment duration and minimal adverse effects. Although pharmacological treatment and intervention are already present in the market, they are far behind from achieving a balanced state and have brought the treatment of UF to a rough path. Botanical drugs are always preferrable by many patients due to their affordability and they are associated with lesser side effects. Therefore, researchers have been investigating various botanical drugs that could be used as an alternative for UF. As UF remains a significant health condition for many women all over the world, the discovery of an effective, safe, and less costly treatment could greatly benefit the society. However, a validated testing protocol is needed to standardize the active constituents in the botanical drug products for use as an anti-UF treatment in ensuring a reproducible therapeutic effect.

## Abbreviations

BMP, bone morphogenetic proteins; ECM, extracellular matrix; ER, estrogen receptor; FOXO, forkhead box O; HuLM, human leiomyoma; IGF-I, insulin-like growth factor-I; MMP, metalloproteinase; NF-κB, nuclear factor kappa B; PKC, protein kinase C; PPARγ, peroxisome proliferator-activated receptor-gamma; TGF, transforming growth factor; TIMP, tissue inhibitor of metalloproteinase; TSC2, tuberous sclerosis complex 2; VEGF, vascular endothelial growth factor.
